# Supplementary material for: Mutational Switch-Backs Can Accelerate Evolution of Francisella to a Combination of Ciprofloxacin and Doxycycline
Source: Front Microbiol. 2022 May 9;13:904822. doi: 10.3389/fmicb.2022.904822 (PMC9125183; doi:10.3389/fmicb.2022.904822)
Supplement: Supplementary file 6 [file Data_Sheet_1.PDF]

Supplementary Material

Supplementary text S1

Biofilm assays:

Biofilm formation was assayed in 100 mm petri dishes. Protocol was adapted from Kumar and Spiro (Kumar and Spiro, 2017). Wild type LVS, FupA/B^{R343I} and TolC^{I436T} were grown to saturation in BHI + 0.1% L-cysteine-hydrochloride at 37°C with shaking at 225 rpm. 10 ml BHI + 0.1% L-cysteine-hydrochloride was added to 100mm x 20mm petri dishes and the dishes were inoculated with 100 µl of saturated culture of the respective strains. Petri dishes were incubated at 37°C without shaking for 48 hrs. Planktonic cultures were decanted and dishes were washed three times with diH_2O. 15 ml of 0.1% crystal violet solution was added to the decanted petri dishes and incubated at room temperature for 20 minutes. Crystal violet was decanted and dishes were washed three times with diH_2O. Dishes were allowed to air dry for 30 minutes. 2 ml 95% ethanol was added to the petri dishes for solubilization of biofilm and measurement was made by reading absorbance at 595 nm. No significant difference in biofilm formation was detected between the wild type and Fup^{R343I} mutant.

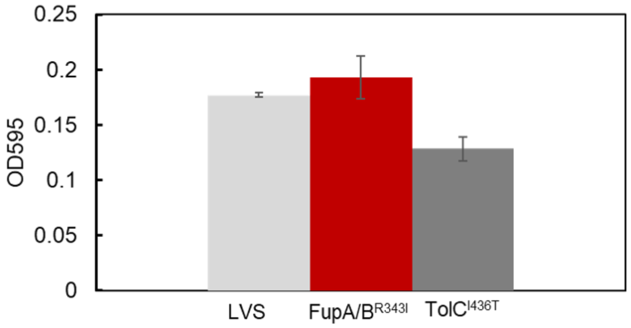

Fig. S1: Biofilm formation in LVS and engineered mutants, FupA/B^{R343I} and TolC^{I436T}. No significant difference observed between amount of biofilm formed in the wild type and FupA/B mutant.

Conserved order of appearance of mutations:

Five replicate populations undergoing combinatorial evolution to both drugs were sampled during the course of evolution and daily samples were sequenced to identify the order and the frequency of mutations involved in adaptation. The four mutational categories implicated in resistance and observed in both, mono- and sequentially-evolved populations were also observed during combinatorial evolution and showed a very consistent order of appearance across replicate populations (Fig. S2).

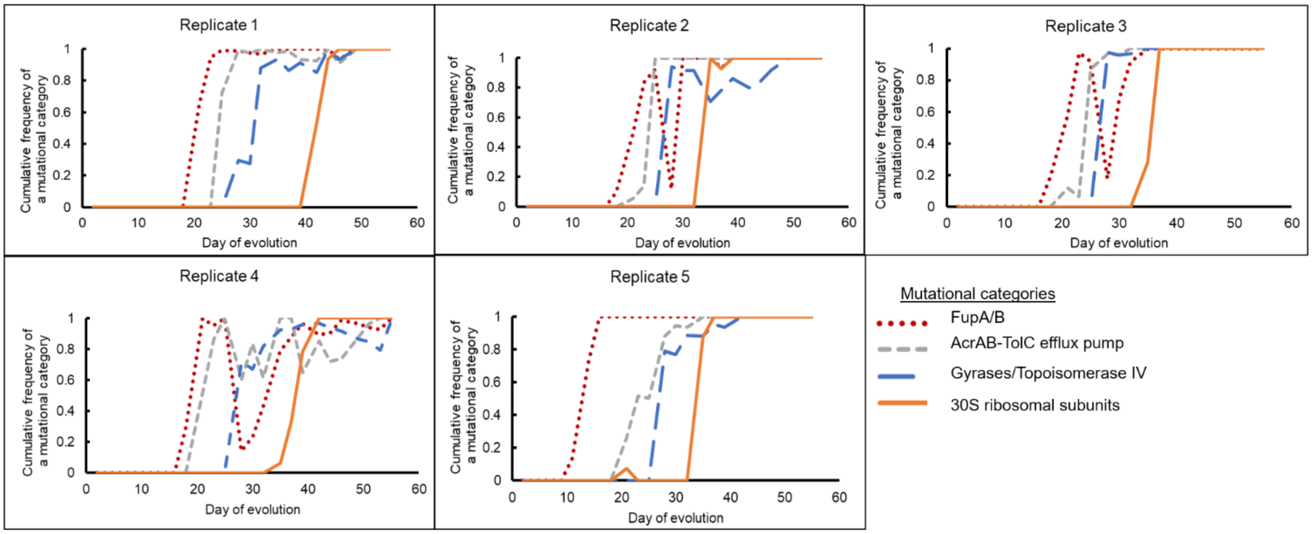

Fig. S2: Consistent parallel evolution observed among replicate populations undergoing combinatorial selection. Each color represents one mutational category as shown in the legend. The Y-axis represents the cumulative frequency of all mutant alleles within that category. The order in which mutations within a category arise is highly conserved such that the first mutants always arise in FupA/B (red dotted), followed by the AcrAB-TolC efflux system (grey dashed), followed by the gyrases or topoisomerases (blue) and the final category is the 30S ribosomal subunits (orange). This order is significant since it plays a role in the alternating evolutionary leapfrogging strategy used by this organism to rapidly evolve resistance to both drugs.

Table S1: End point isolates obtained after mono-selection, MIC values and mutant alleles identified in genes implicated in resistance

Isolate	Population	Doxycycline MIC in µg/ml	Ciprofloxacin MIC in µg/ml	FupA/B	AcrAB-TolC efflux pump	30S ribosomal subunits	Gyrase and topoisomerase IV
Ancestor		0.25	0.01				
**Doxycycline evolved isolates**							
Doxy-2	D-1	> 8	0.16	fs: stop codon at Aa13	TolC I436T	S10 V59L	
Doxy-3	D-1	> 8	0.08	fs: stop codon at Aa13	TolC I436T	S10 V59L	
Doxy-5	D-1	> 8	0.04	Δ37 bp at nt. -8	TolC I436T	S10 V59L	
Doxy-10	D-3	> 8	0.16	D417N	FTL_1107 L405R	S10 V59L	

Doxy-11	D-3	> 8	0.16	D417N	FTL_1107 L405R	S10 V59L	
Doxy-13	D-3	8	0.02	G505E	FTL_1107 L405R	S10 V59L	
Doxy-19	D-5	> 8	0.16	fs: stop codon at Aa4	TolC G429A, I430R	S10 V59L	
Doxy-20	D-5	8	0.08	fs: 1 addt. Aa at C-term	TolC G429A, I430R	S10 V59L	
Doxy-21	D-5	> 8	0.16	G38*	TolC G429A, I430R	S10 V59L	
Doxy-26	D-7	8	0.16	G38*	FTL_1107 L405R	S10 V59L	
Doxy-28	D-7	> 8	0.16	D417N	FTL_1107 L405R	S10 V59L	
Doxy-29	D-7	> 8	0.16	D417N	FTL_1107 L405R	S10 V59L	
Doxy-35	D-10	> 8	0.16	D417N	AcrA L182F	S10 V59L	
Doxy-36	D-10	> 8	0.16	D417N	AcrA L182F	S10 V59L	
Doxy-37	D-10	8	0.04	D417E	FTL_1107 L405F	S10 V59L	
**Ciprofloxacin evolved isolates**							
Cipro-53	C-2	1	> 1.28	D417N			GyrA T83I
Cipro-54	C-2	0.5	> 1.28	D417N			GyrA T83I
Cipro-55	C-2	0.5	> 1.28	D417N			GyrA T83I
Cipro-56	C-3	0.5	> 1.28	D417N			GyrA T83I
Cipro-57	C-3	1	> 1.28	D417N			GyrA T83I
Cipro-58	C-3	0.5	> 1.28	D417N			GyrA T83I
Cipro-68	C-7	1	> 1.28	fs: stop codon at Aa4			GyrA T83K
Cipro-69	C-7	0.5	> 1.28	D417N			GyrA T83K
Cipro-70	C-7	0.125	1.28				GyrA T83K, D87Y, TopoIV subunitB R585I
Cipro-73	C-8	0.5	> 1.28	D417N			GyrA T83I
Cipro-74	C-8	0.5	> 1.28	D417N			GyrA T83I
Cipro-76	C-9	0.5	> 1.28	D417G			GyrA T83I
Cipro-77	C-9	0.5	> 1.28	D417G			GyrA T83I

Note: This is a list of selective mutations. Refer to Supp. Data Set 1 for complete list of mutations in each genome. fs=frameshift, nt.=nucleotide, Aa=amino acid, addt.=additional, FTL_1107 = TolC homolog
Doxycycline and ciprofloxacin concentrations tested for MIC determination were in 2-fold increments. Thus, the values listed in this table may be greater than the actual MIC within a 2-fold range. Values listed here are a mode of 3 to 9 replicates.

Table S2: End point isolates obtained after sequential selection of doxycycline resistant population, MIC values and mutant alleles identified in genes implicated in resistance

Isolate	Doxycycline MIC in µg/ml	Ciprofloxacin MIC in µg/ml	FupA/B	AcrAB-TolC efflux pump	30S ribosomal subunits	Gyrase and topoisomerase IV
Sequential evolution of Doxy^{R} ancestor						
Condition 1: Evolution to BHIC						
Seq-1	8	0.02	37 bp deletion at nt. -8	TolC I436T	S10 V59L	

Seq-2	4	0.02	fs: 1 addt. Aa at C-term.	TolC I430R, G429A	S10 V59L	
Seq-3	4	0.04	fs: 1 addt. Aa at C-term.	TolC I430R, G429A	S10 V59L	
Seq-4	8	0.04	37 bp deletion at nt. -8	TolC I436T	S10 V59L	
Seq-6	8	0.04	37 bp deletion at nt. -8	TolC I436T	S10 V59L	
Seq-7	4	0.04	A456E	TolC I436T	S10 V59L	
Seq-10	8	0.04	fs: 1 addt. Aa at C-term.	TolC I430R, G429A	S10 V59L	
Seq-11	4	0.04	fs: 1 addt. Aa at C-term.	TolC I430R, G429A	S10 V59L	
Seq-13	8	0.04	37 bp deletion at nt. -8	TolC I436T	S10 V59L	
Seq-14	4	0.01	37 bp deletion at nt. -8	TolC I436T	S10 V59L	
Condition 2: Evolution to 8 µg/ml Doxycycline						
Seq-17	8	0.04	G327C	TolC I430R, G429A	S10 V59L, S9 Q125P	
Seq-18	>8	0.04	G327C	TolC I430R, G429A	S10 V59L, S9 Q125P	
Seq-19	>8	0.08	G327C	TolC I430R, G429A	S10 V59L, S9 Q125P	
Seq-22	8	0.08	Q189E, Y192F, S195A	TolC I430R, G429A	S10 V59L	
Seq-24	8	0.04	Q189E, Y192F, S195A	TolC I430R, G429A	S10 V59L	
Seq-25	>8	0.08	Q189E, Y192F, S195A	TolC I430R, G429A	S10 V59L	
Seq-26	>8	0.02	G327C	TolC I430R, G429A	S10 V59L, S9 Q125P	
Seq-27	>8	0.04	G327C	TolC I430R, G429A	S10 V59L, S9 Q125P	
Seq-29	>8	0.04	G327C	TolC I430R, G429A	S10 V59L, S9 Q125P	
Seq-32	8	0.16	Q189E, Y192F, S195A	TolC I430R, G429A	S10 V59L	
Seq-33	8	0.16	Q189E, Y192F, S195A	TolC I430R, G429A	S10 V59L	
Seq-34	8	0.16	Q189E, Y192F, S195A	TolC I430R, G429A	S10 V59L	
Seq-36	>8	0.04	G327C	TolC I430R, G429A	S10 V59L, S9 Q125P	
Seq-38	>8	0.02	G327C	TolC I430R, G429A	S10 V59L, S9 Q125P	
Seq-40	>8	0.04	G327C	TolC I430R, G429A	S10 V59L, S9 Q125P	
Condition 3: Evolution to ciprofloxacin gradient						
Seq-42	8	>1.28	37 bp deletion at nt. -8	TolC I436T	S10 V59L	GyrA T83I
Seq-43	8	>1.28	37 bp deletion at nt. -8	TolC I436T	S10 V59L	GyrA T83I
Seq-44	8	>1.28	37 bp deletion at nt. -8	TolC I436T	S10 V59L	GyrA T83I
Seq-46	8	>1.28	33 bp deletion at nt. -14	TolC I430R, G429A	S10 V59L	GyrA T83I

Seq-47	8	>1.28	33 bp deletion at nt. -14	TolC I430R, G429A	S10 V59L	GyrA T83I
Seq-49	4	>1.28	fs: 1 addt. Aa at C-term.	TolC I430R, G429A	S10 V59L	GyrA T83K
Seq-52	>8	>1.28	37 bp deletion at nt. -8	TolC I436T	S10 V59L	GyrA T83I
Seq-53	8	>1.28	G505E	TolC homolog L405R	S10 V59L	GyrA T83I
Seq-55	4	>1.28	G505E	TolC homolog L405R	S10 V59L	GyrA T83I
Seq-57	4	>1.28	fs: 1 addt. Aa at C-term.	TolC I430R, G429A	S10 V59L	GyrA T83K
Seq-59	8	>1.28	fs: 1 addt. Aa at C-term.	TolC I430R, G429A	S10 V59L	GyrA T83I
Seq-60	4	>1.28	G38*	TolC I430R, G429A	S10 V59L	GyrA T83K
Seq-61	8	>1.28	37 bp deletion at nt. -8	TolC I436T	S10 V59L	GyrA T83I
Seq-62	8	>1.28	37 bp deletion at nt. -8	TolC I436T	S10 V59L	GyrA T83K
Seq-63	8	>1.28	37 bp deletion at nt. -8	TolC I436T	S10 V59L	GyrA T83I
Condition 4: Evolution to ciprofloxacin gradient in the presence of 8 µg/ml doxycycline						
Seq-67	>8	>1.28	Q189E, Y192F, S195A	TolC I430R, G429A	S10 V59L, S9 Q125P	GyrA T83K
Seq-68	>8	>1.28	Q189E, Y192F, S195A	TolC I430R, G429A	S10 V59L, S9 Q125P	GyrA T83K
Seq-69	>8	>1.28	Q189E, Y192F, S195A	TolC I430R, G429A	S10 V59L, S9 Q125P	GyrA D87Y
Seq-70	>8	>1.28	Q189E, Y192F, S195A	TolC I430R, G429A	S10 V59L, S9 Q125P	GyrA T83K
Seq-71	8	>1.28	Q189E, Y192F, S195A	TolC I430R, G429A	S10 V59L	GyrA T83K
Seq-72	8	>1.28	Q189E, Y192F, S195A	TolC I430R, G429A	S10 V59L	GyrA D87Y
Seq-73	8	>1.28	Q189E, Y192F, S195A	TolC I430R, G429A	S10 V59L	GyrA D87Y
Seq-74	8	1.28	Q189E, Y192F, S195A	TolC I430R, G429A	S10 V59L	GyrA D87Y
Seq-76	8	>1.28	Q189E, Y192F, S195A	TolC I430R, G429A	S10 V59L	GyrA D87Y
Seq-77	>8	>1.28	Q189E, Y192F, S195A	TolC I430R, G429A	S10 V59L	GyrA D87Y
Seq-78	8	>1.28	Q189E, Y192F, S195A	TolC I430R, G429A	S10 V59L	GyrA D87Y
Seq-79	>8	>1.28	G327C	TolC I430R, G429A	S10 V59L, S9 Q125P	GyrA T83K
Seq-80	>8	>1.28	Q189E, Y192F, S195A	TolC I430R, G429A	S10 V59L	GyrA T83I
Seq-82	8	>1.28	Q189E, Y192F, S195A	TolC I430R, G429A	S10 V59L	GyrA T83I
Seq-83	8	>1.28	Q189E, Y192F, S195A	TolC I430R, G429A	S10 V59L	GyrA D87Y
Seq-84	8	>1.28	Q189E, Y192F, S195A	TolC I430R, G429A	S10 V59L	GyrA T83I
Seq-86	>8	>1.28	G327C	TolC I430R, G429A	S10 V59L, S9 Q125P	GyrA D87N
Seq-87	>8	>1.28	G327C	TolC I430R, G429A	S10 V59L, S9 Q125P	GyrA T83I

Seq-89	4	>1.28	G327C	TolC I430R, G429A	S10 V59L, S9 Q125P	GyrA T83I
Seq-90	>8	>1.28	Q189E, Y192F, S195A	TolC I430R, G429A	S10 V59L	GyrA D87N

Note: This is a list of selective mutations. Refer to supplementary Dataset 2 for complete list of mutations in each genome. fs=frameshift, nt.=nucleotide, Aa=amino acid, addt.=additional
Doxycycline and ciprofloxacin concentrations tested for MIC determination were in 2-fold increments. Thus, the values listed in this table may be greater than the actual MIC within a 2-fold range. Values listed here are a mode of 3 to 6 replicates.

Table S3: End point isolates obtained after sequential selection of ciprofloxacin resistant population, MIC values and mutant alleles identified in genes implicated in resistance

**Isolate**	**Doxycycline MIC in µg/ml**	**Ciprofloxacin MIC in µg/ml**	**FupA/B**	**AcrAB-TolC efflux pump**	**30S ribosomal subunit S10**	**Gyrase and topoisomerase IV**
Sequential evolution of Cipro^R ancestor						
Condition 1: Evolution to BHIC						
Seq2-1	0.125	1.28				GyrA T83I, GyrA D87Y, TopoIV B L488I
Seq2-2	0.125	1.28				TopoIV A E125D, GyrA T83I
Seq2-3	0.25	0.64				TopoIV A E125D,GyrA T83I
Seq2-5	0.25	1.28				TopoIV A E125D, GyrA T83I
Seq2-7	0.125	0.64				TopoIV A E125D, GyrA T83I
Seq2-8	0.125	0.64				TopoIV A G81C, GyrA T83I
Seq2-10	0.125	0.64				GyrA T83I, GyrA D87Y, TopoIV B L488I
Seq2-11	0.25	1.28				TopoIV A G81C, GyrA T83I
Seq2-12	0.125	1.28				GyrA T83I, GyrA D87Y, TopoIV B L488I
Seq2-14	0.5	0.64				TopoIV A S83I, GyrA T83I
Seq2-15	0.125	0.64				TopoIV A E125D, GyrA T83I
Seq2-16	1	1.28				TopoIV A E125D, GyrA T83I
Condition 2: Evolution to 1 µg/ml Ciprofloxacin						
Seq2-17	0.25	>1.28				GyrA D87Y, T83I; TopoIV B L488I
Seq2-18	0.125	>1.28				GyrA T83I, GyrB E467D, TopoIV B S447I

Seq2-19	0.125	1.28				Topo IV A E125D, GyrA T83I
Seq2-20	0.25	>1.28				TopoIV A S83I, GyrA T83I, GyrA A119S
Seq2-21	0.25	>1.28				TopoIV A S83I, GyrA T83I, GyrB P746H
Seq2-22	0.125	>1.28				TopoIV A G81C, GyrA T83I, GyrB E467D
Seq2-24	0.125	>1.28				TopoIV A G81C, GyrA T83I, GyrB P746H
Seq2-25	0.25	>1.28				TopoIV A S83I, GyrA T83I, GyrB P746H
Seq2-26	0.25	>1.28				GyrA D87Y, T83I; TopoIV B L488I
Seq2-27	0.125	>1.28				GyrA D87Y, T83I; TopoIV B L488I
Seq2-30	0.25	>1.28				TopoIV A S83I, GyrA T83I, GyrB P746H
Seq2-31	0.125	>1.28				GyrA D87Y, T83I; TopoIV B L488I
Seq2-32	0.5	>1.28	G->T at nt. -59			TopoIV A A120E, GyrA T83I, GyrB P746H
Seq2-33	2	>1.28				GyrA D87Y, T83I; TopoIV B L488I
Seq2-34	2	>1.28	fs: stop codon at Aa 4	AcrB P188L		GyrA T83K
Seq2-35	0.125	1.28				TopoIV A A120E, GyrA T83I, GyrB P746H
Seq2-36	4	>1.28	fs: stop codon at Aa 4	AcrB P188L		GyrA T83K
Condition 3: Evolution to doxycycline gradient						
Seq2-37	8	>1.28	G38*	TolC W213C	S10 V59L	GyrA T83I
Seq2-38	8	>1.28	G38*	TolC W213C	S10 V59L	GyrA T83I
Seq2-39	8	>1.28	G38*	TolC W213C	S10 V59L	GyrA T83I
Seq2-40	>8	>1.28	G38*	TolC W213C	S10 V59L	GyrA T83I
Seq2-42	8	>1.28	G38*	TolC W213C	S10 V59L	GyrA T83I
Seq2-43	8	>1.28	G38*	TolC W213C	S10 V59L	GyrA T83I
Seq2-44	8	>1.28	G38*	TolC W213C	S10 V59L	GyrA T83I
Seq2-45	>8	>1.28	G38*	TolC W213C	S10 V59L	GyrA T83I
Seq2-46	>8	>1.28	G38*	TolC W213C	S10 V59L	GyrA T83I
Seq2-47	>8	>1.28	G38*	TolC W213C	S10 V59L	GyrA T83I
Seq2-48	>8	>1.28	G38*	TolC W213C	S10 V59L	GyrA T83I
Seq2-49	>8	>1.28	G38*	TolC W213C	S10 V59L	GyrA T83I

Seq2-50	>8	>1.28	G38*	TolC W213C	S10 V59L	GyrA T83I
Seq2-52	>8	>1.28	G38*	TolC W213C	S10 V59L	GyrA T83I
Condition 4: Evolution to doxycycline gradient in the presence of 1 μg/ml ciprofloxacin						
Seq2-53	8	>1.28	G38*	TolC W213C	S10 V59L	GyrA T83I
Seq2-54	8	>1.28	G38*	TolC W213C	S10 V59L	GyrA T83I
Seq2-55	8	>1.28	G38*	TolC W213C	S10 V59L	GyrA T83I
Seq2-56	>8	>1.28	G38*	TolC W213C	S10 V59L	GyrA T83I
Seq2-57	>8	>1.28	D417N	TolC Q241K	S10 V59L	GyrA T83I
Seq2-58	>8	>1.28	D417N	TolC Q241K	S10 V59L	GyrA T83I
Seq2-59	>8	>1.28	D417N	TolC Q241K	S10 V59L	GyrA T83I
Seq2-60	>8	>1.28	D417N	TolC Q241K	S10 V59L	GyrA T83I
Seq2-61	>8	>1.28	D417N	TolC W213C	S10 V59L	GyrA T83I
Seq2-63	>8	>1.28	D417N	TolC W213C	S10 V59L	GyrA T83I
Seq2-64	>8	>1.28	D417N	TolC W213C	S10 V59L	GyrA T83I
Seq2-65	>8	>1.28	D417N	AcrA L182F	S10 V59L	GyrA T83I
Seq2-66	>8	>1.28	D417N	AcrA L182F	S10 V59L	GyrA T83I
Seq2-67	>8	>1.28	D417N	AcrA L182F	S10 V59L	GyrA T83I
Seq2-68	>8	>1.28	D417N	AcrA L182F	S10 V59L	GyrA T83I
Seq2-69	>8	>1.28	D417N	AcrA L182F	S10 V59L	GyrA T83I
Seq2-70	>8	>1.28	D417N	TolC W213C	S10 V59L	GyrA T83I
Seq2-71	>8	>1.28	D417N	TolC W213C	S10 V59L	GyrA T83I
Seq2-72	>8	>1.28	D417G	TolC W213L	S10 V59L	GyrA T83I

Note: This is a list of selective mutations. Refer to Supp. DataSet 3 for complete list of mutations in each genome. fs=frameshift, nt.=nucleotide, Aa=amino acid
Doxycycline and ciprofloxacin concentrations tested for MIC determination were in 2-fold increments. Thus, the values listed in this table may be greater than the actual MIC within a 2-fold range. Values listed here are a mode of 3 to 6 replicates.

Table S4: End point isolates obtained after combinatorial selection, MIC values and mutant alleles identified in genes implicated in resistance

Isolate	Population	Doxycycline MIC in μg/ml	Ciprofloxacin MIC in μg/ml	FupA/B	AcrAB-TolC efflux pump	30S ribosomal subunits	Gyrase and topoisomerase IV
Comb-1	CD-1	8	>1.28	D417N	AcrA L182F	S10 V59L	GyrA D87Y
Comb-2	CD-1	>8	>1.28	D417N	AcrA L182F	S10 V59L	GyrA D87Y
Comb-3	CD-1	>8	>1.28	D417N	AcrA L182F	S10 V59L	GyrA D87Y
Comb-4	CD-1	>8	>1.28	D417N	AcrA L182F	S10 V59L	GyrA D87Y
Comb-6	CD-2	>8	>1.28	G38*	FTL_1107 L405F	S10 V59L	GyrA D87Y
Comb-7	CD-2	>8	>1.28	G38*	FTL_1107 L405F	S10 V59L	GyrA D87Y

Comb-8	CD-2	>8	>1.28	G38*	FTL_1107 L405F, TolC E441K	S10 V59L	GyrA D87Y
Comb-9	CD-2	>8	>1.28	G38*	FTL_1107 L405F	S10 V59L	GyrA D87Y
Comb-12	CD-3	8	>1.28	S332I, R343I	TolC I436T	S10 V59L	GyrA D87Y
Comb-13	CD-3	>8	>1.28	R343I	TolC I436T	S10 V59L	GyrA D87Y
Comb-14	CD-3	>8	>1.28	S332I, R343I	TolC I436T	S10 V59L	GyrA D87Y
Comb-16	CD-4	>8	>1.28	fs: stop codon at Aa 172	FTL_1107 S166R, AcrA F268L	S10 V59L	GyrA T83I
Comb-18	CD-4	>8	>1.28	fs: stop codon at Aa 172	FTL_1107 S166R, AcrA F268L	S10 V59L	GyrA T83I
Comb-19	CD-4	>8	>1.28	fs: stop codon at Aa 172	AcrA F268L	S10 V59L	GyrA D87Y
Comb-21	CD-5	>8	>1.28	R343I	AcrB S248I, TolC I436T	S10 V59L	GyrA D87Y
Comb-22	CD-5	>8	>1.28	R343I	AcrB S248I, TolC I436T	S10 V59L	GyrA D87Y
Comb-24	CD-5	>8	>1.28	R343I	AcrB S248I, TolC I436T	S10 V59L	GyrA D87Y

Note: This is a list of selective mutations. Refer to Supp. DataSet 4 for complete list of mutations in each genome. fs=frameshift, Aa=amino acid, FTL_1107=TolC homolog.
Doxycycline and ciprofloxacin concentrations tested for MIC determination were in 2-fold increments. Thus, the values listed in this table may be greater than the actual MIC within a 2-fold range. Values listed here are a mode of 3 to 9 replicates.

Kumar, S. and Spiro, S. (2017) ‘Environmental and Genetic Determinants of Biofilm Formation in Paracoccus denitrificans’, *mSphere*, 2(5). doi: 10.1128/mspheredirect.00350-17.
